# Stem cell factor produced by tumor cells expands myeloid-derived suppressor cells in mice

**DOI:** 10.1038/s41598-020-68061-8

**Published:** 2020-07-09

**Authors:** Wei-Chen Lee, Pao-Yueh Hsu, Hsiu-Ying Hsu

**Affiliations:** Division of Liver and Transplantation Surgery, Department of General Surgery, Chang-Gung Memorial Hospital, Chang-Gung University College of Medicine, 5, Fu-Hsing Street, Kwei-Shan Township, Taoyuan, Taiwan

**Keywords:** Gastroenterology, Medical research

## Abstract

Immunotherapy is a novel treatment approach for cancers; however, its therapeutic effects are impeded by myeloid-derived suppressor cells (MDSCs). This study aimed to determine how MDSCs are expanded in cancer hosts. MDSCs were positive for Gr-1 and CD11b. Hepa1-6 hepatoma cells, EL4 lymphoma cells, and mice bearing Hepa1-6 hepatoma or lymphoma were examined. Following the inoculation of Hepa1-6 cells into the flanks of mice, a linear correlation was evident between the frequency of MDSCs in the spleen and tumor sizes. MDSC numbers diminished gradually and returned to the normal level within 3 weeks if the tumors were excised. To identify the cytokines produced by tumor cells that allowed expansion of MDSCs, cytokines in Hepa1-6 cell culture medium and murine serum were examined using a cytokine array. Stem cell factor (SCF) was implicated as the relevant cytokine. When recombinant SCF was added to the spleen cell culture medium, MDSC expansion could occur. In the presence of c-kit blockade, this effect of SCF was partially reversed. In conclusion, MDSCs can be expanded in tumor cells in a process that involves SCF released by tumor cells.

## Introduction

The immune system is the most important protective means used by a host to defend against foreign invaders and cancer development. Cancer cells are formed as a consequence of enhanced or aberrant expression of oncogenes or loss of tumor suppressor genes. Thereafter, cancer cells express tumor-specific or tumor-associated antigens, which can be applied to trigger antitumor immunity^1^.

Several decades ago, immunotherapy was recognized as an attractive and novel strategy to treat cancer^2^. Cytokine-based immunotherapy for cancer proved disappointing. However, cancer immunotherapy regained enthusiasm with the demonstrated potential of dendritic cells (DCs) in cancer treatment^3–7^. Tumor-specific or tumor-associated antigens might be recognized by antigen-presenting cells, in turn triggering T-lymphocyte mediated anti-cancer immunity. Recently, immune checkpoint inhibitors have been applied to treat malignant tumors. A substantial survival benefit has been reported in some patients^8,9^. The findings to date have implicated immunotherapy as an emerging and potential treatment for cancers.

DC-based immunotherapy has a great potential for the successful treatment of cancer. Since the 1990s, DCs have been applied to treat advanced malignant diseases, including B-cell lymphoma, renal cell carcinoma, prostate cancer, colorectal carcinoma, hepatocellular carcinoma (HCC), and pancreatic cancer^10–16^. While tumor response to DC treatment occurs, the response rate has been low (15–40%)^12,14,17^. The experience of clinical trials has shown that a host’s anti-cancer immunity is already suppressed in advanced cancers, which lessens the chance of successful immunotherapy. In an animal study, DCs were effective in treating small HCC tumors, but could only slow down the growth of large HCC tumors^18^. There is no doubt that anti-cancer immunity is suppressed when the malignant tumors progress^19–21^. Thus, to achieve successful immunotherapy for cancer, immunosuppressive factors must be eliminated.

A group of cells that express Gr-1 and CD11b appear in the spleen and lymph nodes in tumor-bearing mice^22,23^. This group of cells is termed myeloid-derived suppressor cells (MDSCs)^24^. The mechanisms used by MDSCs to suppress other immune cells have been explored. MDSCs can induce T-cell tolerance, activate regulatory T-cells, suppress DC differentiation, and induce anergy of natural killer (NK) cells^25–28^. All these effects are detrimental to immunotherapy for a host with advanced malignancy.

In humans, MDSCs have been identified in patients with advanced cancers^28,29^. Many factors may expand MDSC^30–32^, but there is no direct evidence. This study examined the possible factors associated with expansion of MDSCs. The findings could inform the design of strategies for treatment of malignancies.

## Results

### MDSCs in the spleen of tumor-bearing mice

**Figure 1:**
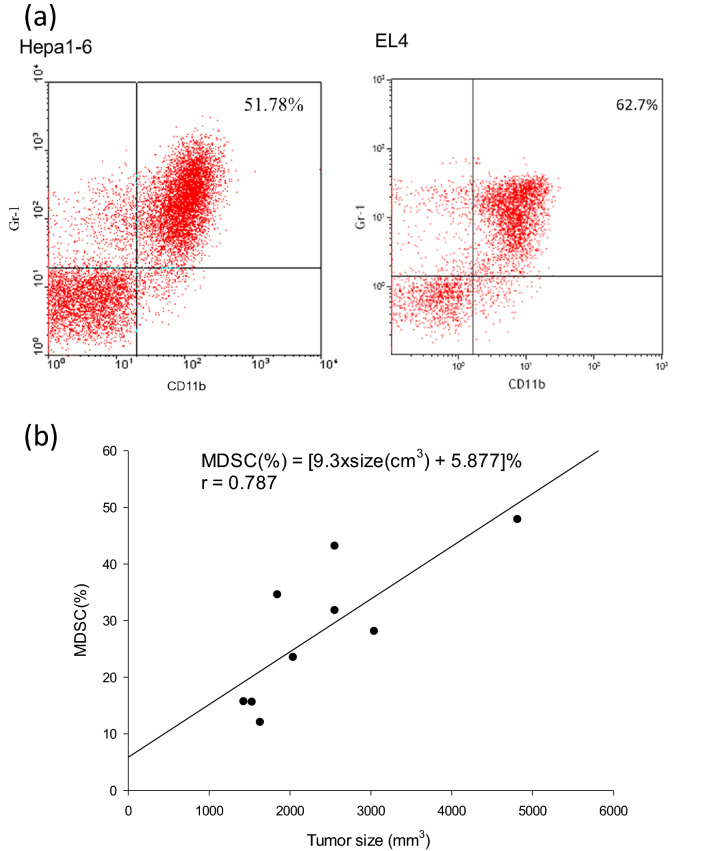
MDSCs in tumor-bearing mice. (**a**) Representative MDSCs expressing Gr-1 and CD11b in the spleen cells from B10 mice bearing Hepa1-6 tumors and B6 mice bearing EL4 tumors. (**b**) The frequency of MDSCs in 10 B10 mice was correlated to tumor volume. A linear correlation was evident between MDSC frequency and tumor volume. The percentage of MDSCs was calculated as [9.3 × tumor size (cm^3^) + 5.877]%.

To determine MDSC induction, spleens were harvested from B10 mice harboring Hepa1-6 tumors and from B6 mice harboring EL4 tumors, when the tumors were approximately 15 mm in diameter. A suspension of single spleen cells was prepared. The spleen cells were stained using phycoerythrin (PE)-conjugated anti-Gr-1 and fluorescein isothiocyanate (FITC)-conjugated anti-CD11b, and analyzed by flow cytometry. Gr-1^+^CD11b^+^ cells were detected in the tumor-bearing mice (Fig. 1a). To determine whether the population of Gr-1^+^CD11b^+^ cells was correlated with tumor size, 10 B10 mice bearing different-sized Hepa1-6 tumors were sacrificed and their spleens were harvested. The spleen cells were stained by Gr-1 and CD11b. Gr-1^+^CD11b^+^ MDSCs were linearly correlated with tumor size. The correlation allowed the percentage of MDSCs to be calculated as [9.3 × tumor size (cm^3^) + 5.877]% (Fig. 1b).

### Reversal of MDSC frequency by removal of tumors

B10 mice were inoculated in their flank with Hepa1-6 tumor cells. When the diameters of the tumors approached 15 mm, the tumors were excised completely. The spleens were harvested at postoperative week 1, 3, and 5. The frequency of MDSCs was 2.53 ± 1.44% in naïve mice (n = 11). According to the above equation relating MDSC frequency and tumor size, the frequency of MDSC in the spleen was 22.2% for 15 mm-diameter tumors. After excision of the tumors, the frequency of MDSCs in the spleen was 5.23 ± 2.42% (n = 3) at postoperative week one (p = 0.045, compared to naïve mice), 2.97 ± 1.86% (n = 3) at postoperative week 3 (p = 0.663, compared to naïve mice), and 3.12 ± 1.05% (n = 2) at postoperative week 5. These findings indicated that MDSCs were maintained by tumors. In this tumor model, the frequency of MDSCs diminished gradually and returned to normal within 3 weeks after the tumors were excised.

### Expansion of MDSCs by conditioned medium

**Figure 2:**
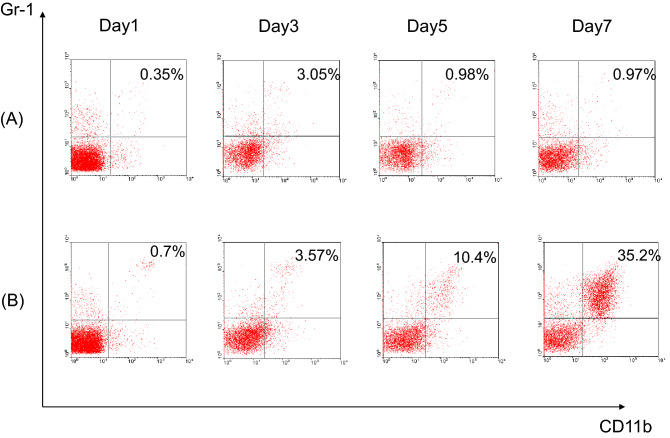
Representative MDSCs expanded by the conditional medium. Naïve spleen cells were cultured in (**A**) RPMI 1640 with 10% FCS and (**B**) conditional medium collected from Hepa1-6 cell culture. Gr-1^+^CD11b^+^ cells were increased beginning on day 5 and were very prevalent by day 7 when the cells were cultured in conditional medium.

The basis of the expansion of MDSCs is unclear. The finding that tumor removal could eliminate MDSCs in the spleen indicated that some cytokines must be released by cancer cells to induce MDSCs. To confirm that such eligible cytokines were produced by cancer cells, conditional medium collected from Hepa1-6 cell culture was used to culture naïve spleen cells. Gr-1^+^CD11b^+^ MDSCs were analyzed by flow cytometry on day 1, 3, 5, and 7. Gr-1^+^CD11b^+^ cells were not increased in the first 3 days. However, beginning on day 5, increased numbers of Gr-1^+^CD11b^+^ were evident (14.11 ± 4.71% versus 2.21 ± 1.06% for the control, p < 0.001). On day 7, the proportion of Gr-1^+^CD11b^+^ cells among the total cells had increased significantly (23.97 ± 10.43% versus 2.66 ± 1.71% for the control, p = 0.006). These results clearly showed that cytokine(s) capable of stimulating expansion of MDSCs were produced by cancer cells, and that Gr-1^+^CD11b^+^ MDSCs could be expanded during culture in the conditioned medium (Fig. 2).

### Release of cytokines by tumor cells

**Figure 3:**
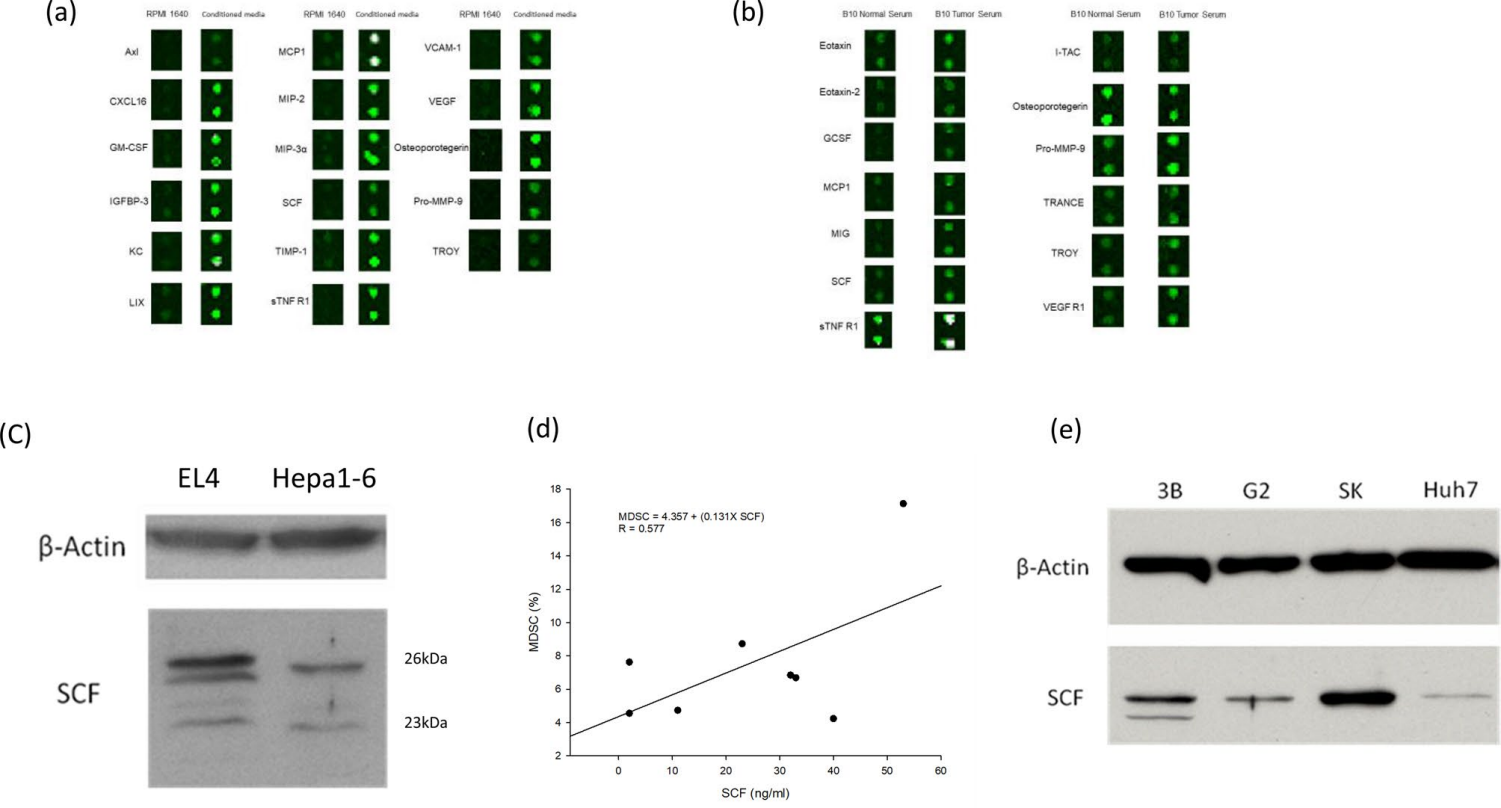
Eligible cytokines contributing to expansion of MDSCs. (**a**) Overproduced cytokines were identified by cytokine antibody array in the conditioned medium. They included GM-CSF, SCF, MCP-1, sTNF-R1, and pro-MMP-9. (**b**) Cytokines in the serum from naïve and Hepa1-6 tumor-bearing mice were identified by cytokines array. SCF, MCP-1, G-CSF, sTNF-R1, and pro-MMP-9 were increased in the serum of tumor-bearing mice. (**c**) Western blotting showed soluble and membrane-bound forms of SCF produced by Hepa1-6 cells and EL4 cells (**d**). When SCF was measured by ELISA, the serum level of SCF tended to correlate with MDSC frequency in tumor-bearing mice: MDSC (%) = 4.357 + 0.131 × SCF (ng/ml) (r = 0.577). (**e**) Western blotting also showed that human HepG2, 3B, SK, and Huh7 cells produced SCF.

To further identify the cytokines released by cancer cells, the conditioned medium was analyzed using a cytokine array. Compared with RPMI-1640 containing 5% fetal calf serum (FCS), several cytokines were detected at higher levels in the conditioned medium. These included granulocyte–macrophage colony stimulating factor (GM-CSF), stem cell factor (SCF), monocyte chemoattractant protein 1 (MCP-1), soluble tumor necrosis factor-receptor 1 (sTNF-R1), pro-matrix metalloproteinase-9 (pro-MMP-9), and osteoporotegerin (Fig. 3a). Cytokines in the serum from naïve and Hepa1-6 tumor-bearing mice were identified using the cytokine array. The expression levels of several cytokines including SCF, MCP-1, granulocyte-colony stimulating factor (G-CSF), sTNF-R1, and pro-MMP-9 were increased in the serum of tumor-bearing mice (Fig. 3b). SCF, MCP-1, sTNF-R1, and pro-MMP-9 were increased in both the conditioned medium and serum. When SCF, MMP-9, or SCF and MMP-9 was added to spleen cell culture medium, MDSCs only appeared in the medium containing SCF. Therefore, SCF was considered as the most eligible cytokine capable of inducing MDSCs in conditioned medium or serum of tumor-bearing mice among the common cytokines. To further confirm that SCF was actually released by tumor cells, total protein was extracted from Hepa1-6 and western blotting was performed. Hepa1-6 cells produced soluble and membrane-bound forms of SCF (Fig. 3c). Furthermore, when serum SCF was measured by ELISA, the serum level of SCF tended to correlate with MDSC frequency in tumor-bearing mice [MDSC (%) = 4.357 + 0.131 × SCF (ng/ml); r = 0.577] (Fig. 3d). To determine whether human tumor cells could produce SCF similar to murine tumor cells, total protein was extracted from human Hep G2, 3B, SK, and Huh7 cells and western blotting was performed. These human tumor cells also produced SCF (Fig. 3e).

### *Expansion of MDSC by SCF *in vitro

**Figure 4:**
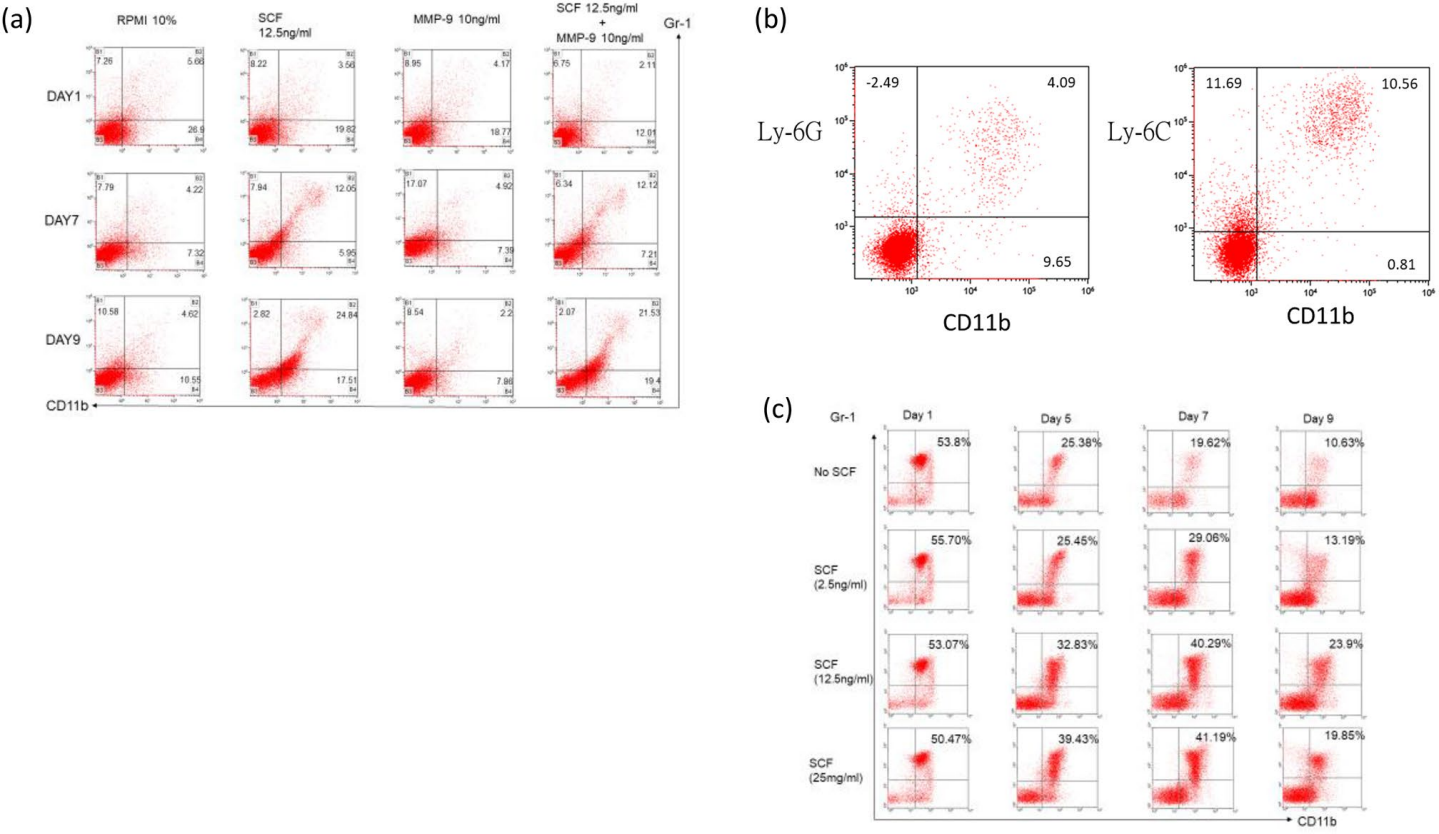
Representative MDSCs expanded and maintained by SCF. (**a**) Gr-1^+^CD11b^+^ cells appeared from day 7 of culture when 12.5 ng/ml recombinant mouse SCF was added to the culture medium. On day 9 of culture, approximately 25% of the cultured spleen cells were Gr-1^+^CD11b^+^. Gr-1^+^CD11b^+^ cells were not increased when MMP-9 was added to the culture medium. (**b**) When large Gr-1^+^CD11b^+^ cells were gated, Ly-6C^+^ cells were more prevalent than Ly-6G^+^ cells. (**c**) The spleen cells harvested from tumor-bearing mice were cultured in RPMI-1640 and different doses of SCF were added. Gr-1^+^CD11b^+^ cells were diminished gradually if SCF was not added to the culture medium. The population of Gr-1^+^CD11b^+^ cells could be maintained when SCF (≥ 12.5 ng/ml) was added.

To determine whether SCF or MMP-9 was the actual cytokine that stimulated expansion of MDSCs, 12.5 ng/ml of recombinant mouse SCF and/or 10 ng/ml of MMP-9 were added to the B10 spleen cell culture medium. Gr-1^+^CD11b^+^ cells detected by flow cytometry were only evident in the culture supplemented with SCF beginning at day 7 of culture (14.85 ± 4.03% versus 5.10 ± 1.35% in RPMI and 4.77 ± 1.76%, p = 0.002). On day 9 of culture, 26.66 ± 8.25% of the cultured spleen cells were Gr-1^+^CD11b^+^ (Fig. 4a). Further analysis of cells by staining with Ly-6C and Ly-6G showed that the frequency of CD11b^+^Ly-6C^+^ cells was higher than that of CD11b^+^Ly-6G^+^ cells (Fig. 4b). The immune suppressive function of these CD11b^+^Ly-6G^+^ and CD11b^+^Ly-6C^+^ cells was determined on the basis of arginase activity. Both the CD11b^+^Ly-6G^+^ and CD11b^+^Ly-6C^+^ cells displayed higher arginase activity than splenocytes (7.810 ± 6.211 U/l and 5.271 ± 2.794 U/l, respectively, versus 0.291 ± 0.426 U/l, p = 0.011). We further examined whether SCF was necessary to maintain Gr-1^+^CD11b^+^ cells. Spleen cells harvested from tumor-bearing mice were maintained in RPMI-1640 containing different concentrations of SCF. Gr-1^+^CD11b^+^ cell numbers diminished gradually if no SCF was present in the culture medium. When SCF was added (≥ 12.5 ng/ml), the population of Gr-1^+^CD11b^+^ cells could be maintained. At 7 days of culture in the presence of SCF, the frequency of Gr-1^+^CD11b^+^ cells were maintained at 38.79 ± 11.36%, compared to 17.46 ± 5.29% for the control (p = 0.333). The respective vales after 9 days of culture with SCF was 22.54 ± 5.09% and 7.15 ± 3.619% (p = 0.003, Fig. 4c).

### Suppression of T-cell proliferation by MDSCs

**Figure 5:**
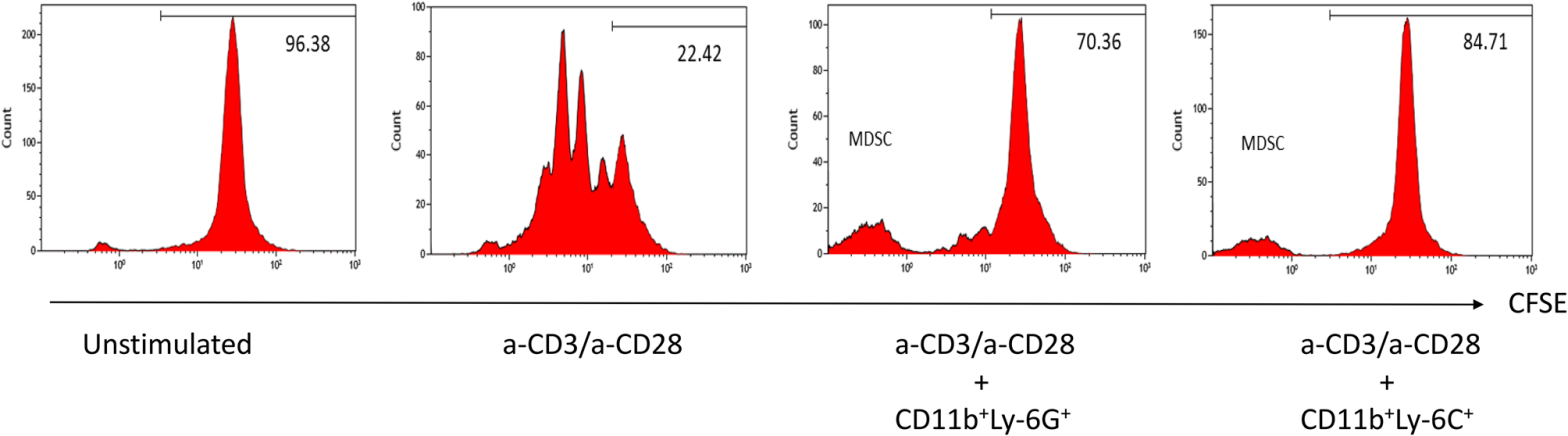
T-cell proliferation is suppressed by MDSCs. B10 T-cells were stained by carboxyfluorescein succinimidyl ester and activated by anti-CD3/anti-CD28. The proliferation of T-cells was suppressed by MDSC at 1:1 of T-cells and CD11b^+^Ly-6G^+^ or CD11b^+^Ly-6C^+^ MDSC.

To determine the suppressive effects of MDSCs cultured in the presence of SCF, Gr-1^+^CD11b^+^ MDSCs were isolated using a commercial kit and sub-grouped into CD11b^+^Ly-6G^+^ and CD11b^+^Ly-6C^+^ cells. These cells were added to a mixed lymphocyte reaction of T-cells activated by anti-CD3/anti-CD28. The proliferation of T-cells was suppressed by MDSCs using a 1:1 ratio of T-cells and CD11b^+^Ly-6G^+^ or CD11b^+^Ly-6C^+^ MDSC (Fig. 5).

### Blocking SCF decreases the frequency of MDSCs

**Figure 6:**
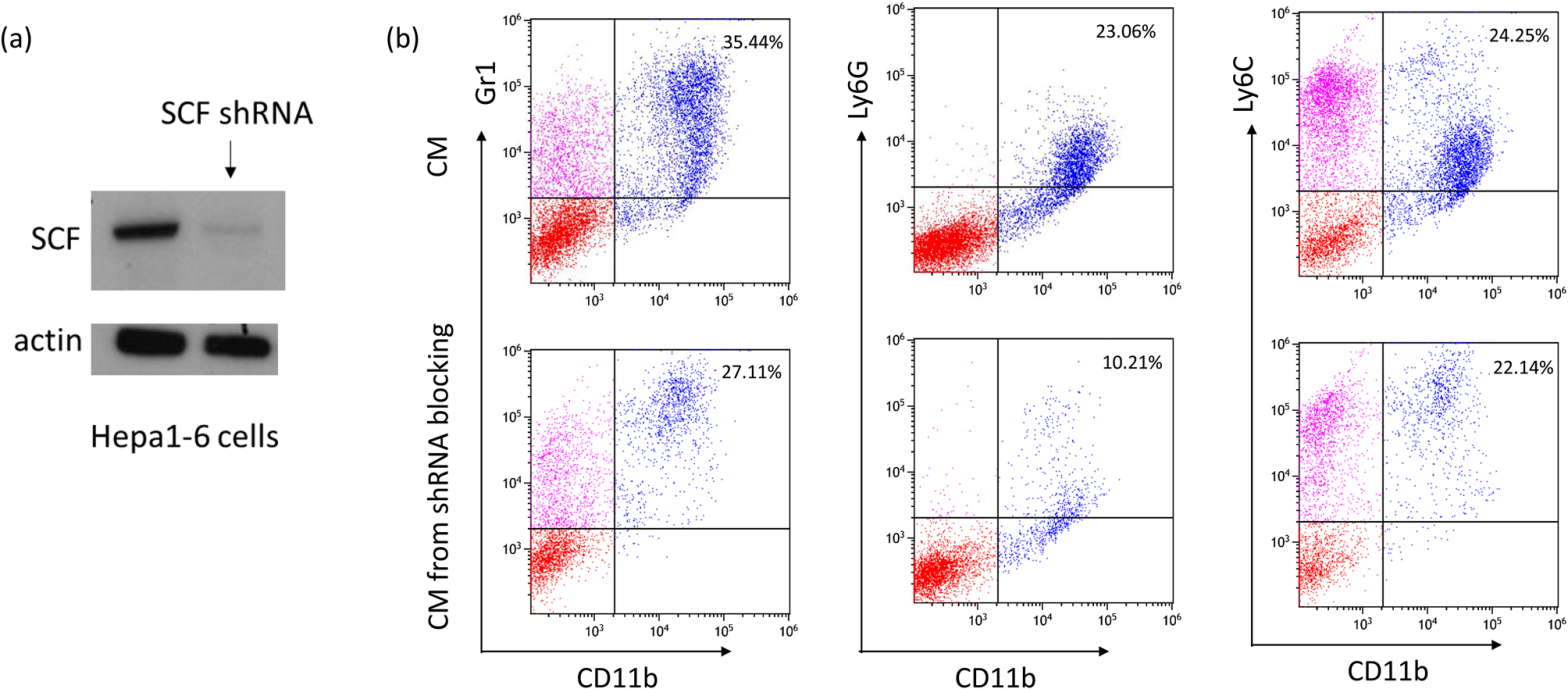
Representative Hepa 1–6 transfection with shRNA of SCF. (**a**) The expression of SCF was effectively blocked by pENTR/H1/TO shRNA of SCF. (**b**) The frequency of MDSCs decreased when the spleen cells were cultured in the conditional medium collected from Hepa1-6 cells blocked by pENTR/H1/TO shRNA.

**Figure 7:**
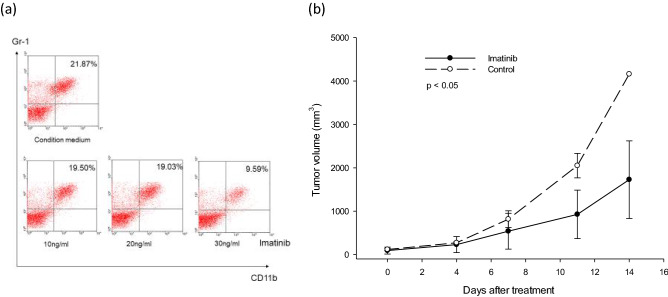
Representative decrease in the number of MDSCs by Imatinib. (**a**) The spleen cells harvested from B10 mice were maintained in conditioned medium for 7 days to expand Gr-1^+^CD11b^+^ cells. Different doses of Imatinib (10, 20, and 30 ng/ml) were added to block the effect of SCF. The frequency of Gr-1^+^CD11b^+^ cells was decreased when 30 ng/ml Imatinib was added. (**b**) When EL4 tumors on the back of B6 mice was 5 × 5 mm in diameter, the mice were treated with Imatinib. The tumor growth in Imatinib-treated mice was slower than in control mice. On day 11 of treatment, the tumor volume was 927.7 ± 558.5 mm^3^ in the mice treated with Imatinib, compared to 2050.4 ± 282.7 mm^3^ in control mice (p = 0.023).

To determine whether blocking of SCF would decrease the frequency of MDSCs, Hepa1-6 cells were transfected with pENTR/H1/TO short hairpin RNA (shRNA) targeting SCF. The conditional medium used to culture MDSCs was collected and analyzed. The expression of SCF could be effectively blocked by the silencing of SCF using pENTR/H1/TO shRNA (Fig. 6a). The frequency of MDSCs decreased when the spleen cells were cultured in the conditional medium collected from Hepa1-6 cells transfected with pENTR/H1/TO shRNA (Fig. 6b). As c-kit is the receptor of SCF and Imatinib blocks c-kit, we further examined whether Imatinib could block SCF and decrease the frequency of MDSCs. Spleen cells harvested from B10 mice were cultured in conditioned medium for 7 days to expand MDSCs. Subsequently, different doses of Imatinib (10, 20, and 30 ng/ml) were added to the culture medium. The frequency of Gr-1^+^CD11b^+^ cells tended to decrease using 20 or 30 ng/ml Imatinib (21.54 ± 2.68% and 20.07 ± 9.43% compared to 28.92 ± 6.97% for the control, p = 0.099) (Fig. 7a). To examine the effects in vivo, Imatinib (25 mg) was injected into peritoneum of tumor-bearing mice each day for 9 days, and tumor growth was measured. When the EL4 tumors on the backs of B6 mice were 5 × 5 mm in diameter, the mice were treated with Imatinib. The tumor growth in Imatinib-treated mice was slower than in control mice. On day 11 of treatment, the tumor volume was 927.7 ± 558.5 mm^3^ in the mice treated with Imatinib, compared to 2050.4 ± 282.7 mm^3^ in control mice (p = 0.023, Fig. 7b).

### Survival benefit of splenectomy for tumor-bearing mice

**Figure 8:**
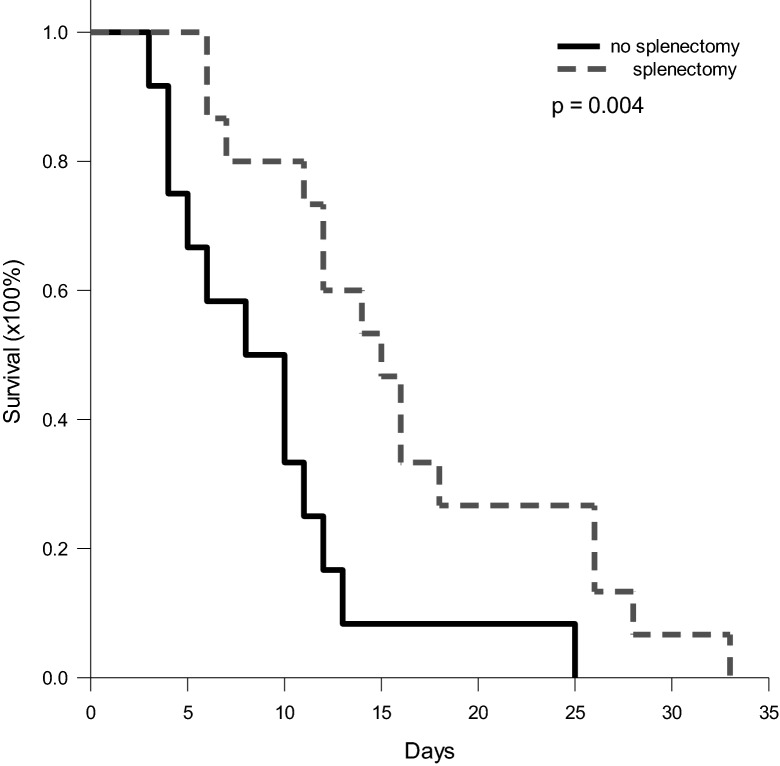
Survival rate of mice bearing Hepa1-6 tumors after splenectomy. Twelve tumor-bearing mice received a splenectomy. Their survival was significantly prolonged compared to that of mice without splenectomy (16.4 ± 8.36 versus 9.25 ± 6.02 days, p = 0.020).

Most of the Gr-1^+^CD11b^+^ MDSCs accumulated in the spleens in the tumor-bearing mice. Removal of MDSCs might be beneficial for host survival. To assess the immunosuppressive effect of MDSCs in vivo, the spleens of tumor-bearing mice were excised to examine whether the survival of the mice could be prolonged. Twenty-four mice bearing tumors 15 mm in diameter were divided into two groups. Splenectomy was performed in 12 of the mice. The survival of the mice who received splenectomy was prolonged compared to the mice without splenectomy (16.4 + 8.36 vs. 9.25 + 6.02 days, p = 0.020) (Fig. 8).

## Discussion

MDSCs are a type of immunosuppressive cell in tumor-bearing mice. In this study, the frequency of MDSCs in the spleen was correlated with tumor size. In Hepa1-6 mouse hepatoma model, the frequency of MDSCs in the spleen could be calculated. Diaz-Montero et al. reported elevated numbers of MDSCs in the circulating peripheral blood of cancer patients compared to health volunteers^29^. In human HCC, MDSCs could be detected in peripheral blood and displayed immunosuppressive activity by inducing regulatory T-cells^28^. Gabitass et al. also mentioned that the frequency of MDSCs was increased in pancreatic, gastric, and esophageal cancers^33^. The present findings further show that the frequency of MDSCs can be correlated with the tumor burden in the hosts.

MDSCs are induced and maintained by tumors. Presently, when tumors that had developed in the flank of mice injected with Hepa1-6 hepatoma cells were completely resected, the frequency of MDSCs returned to normal within 3 weeks. Salrador et al. demonstrated that the cell population in the spleen returned to normal following excision of fibrosarcoma in the mice^34^. These results imply the existence of a paracrine effect, where particular cytokines must be released by the tumor cells to maintain the population of MDSCs. In vitro, MDSCs appeared when naïve spleen cells were cultured in Hepa1-6 conditioned medium for 7 days. Particular cytokines capable of inducing MDSCs must be produced and released into the cell culture medium from tumors. However, the identity of these particular cytokines has been unclear. In this study, several candidates were identified using a cytokine assay.

SCF was one of the cytokines capable of stimulating MDSC expansion. The cytokine assay revealed that several common cytokines were overproduced in the supernatant of cultured tumor cells as well as in the serum of tumor-bearing mice. Among these common overproduced cytokines, SCF was the most eligible cytokine to induce MDSCs. SCF appeared in the supernatant of Hepa1-6 cell cultures but not in medium of naïve cell cultures. SCF was overproduced in the serum of tumor-bearing mice, compared with the serum from naïve mice. SCF is a hematopoietic cytokine that promotes the survival, proliferation, and differentiation of hematopoietic cells. Western blot analysis also showed that Hepa1-6 could produce membrane-bound and soluble SCF. Direct measurement of SCF in the serum of Hepa1-6 tumor-bearing mice correlated the level of serum to tumor size. We further demonstrated that SCF could induce MDSCs. Supplementation of the RPMI-1640 medium with ≥ 12.5 ng/ml SCF resulted in the induction of a significant population of Gr-1^+^CD11b^+^ MDSCs after 9 days of culture. Eligible cytokines that could induce MDSCs have been reviewed^31^. However, no direct evidence that a single cytokine can induce MDSCs has been previously published. Pan et al. mentioned that block SCF enhanced immune-enhancing cancer therapy^35^. The present findings provide direct evidence that SCF can stimulate expansion of MDSCs.

C-kit is a cell surface receptor of SCF. Imatinib blocks c-kit. In the experiment in which SCF or conditioned culture medium were present during spleen cell culture, MDSC expansion was evident. When Imatinib was added to block c-kit, the SCF-c-kit downstream pathway could not be activated and the MDSC population decreased. When SCF was directly blocked by shRNA, the frequency of MDSCs cultured using conditional medium was decreased. These results provided conformation that SCF was necessary to maintain MDSCs. Several cancer therapy strategies have been employed to block or diminish MDSCs^36^. In one study, the Toll-like receptor ligand CpG was used to block the suppressive effect of MDSC on T-cells. The tumor size in tumor-bearing mice could be reduced significantly^37^. Gemcitabine in cancer therapy can reduce the number of MDSCs, increase the antitumor activity of CD8^+^ T-cells, and activate NK cells^38^. In this study, the tumor growth rate was decreased when EL4 tumor-bearing mice were treated by Imatinib. The collective results indicate that tumor sizes can be reduced when the MDSC population is decreased. However, the tumors were not treated successfully. The findings imply that directly targeting SCF might be helpful for cancer treatment.

MDSCs in tumor-bearing hosts are not only immunosuppressive cells. They also contributes to survival. In our animal model, when the spleen was removed, the survival of the mice was significantly prolonged. The spleen is the largest immune organ and plays a role in defense to infection or foreign antigens. However, many MDSCs accumulate in the spleen when a host harbors a large tumor. In this setting, the spleen is no more an immune-defense organ and contrarily contributes to immunosuppression. Removal of the spleen to diminish MDSCs and prolong survival clearly demonstrates that removal of the spleen removes the immunosuppressive effect of MDSC in tumor-bearing hosts. Hence, diminishing MDSCs could be a valuable strategy in the treatment of cancer.

In conclusion, MDSCs are immunosuppressive cells that compromise the immunity in a tumor-bearing individual. The SCF cytokine that is released by tumor cells stimulates expansion of MDSCs. Blocking the SCF pathway may decrease the population of MDSCs and could be valuable in cancer treatment.

## Materials and methods

### Mice

Six-to-eight-week-old male C57BL/10J (B10; H-2^b^, I-A^b^) mice and C57BL/6J (B10; H-2^b^, I-A^b^) mice were purchased from the Animal Laboratory of National Institute (Taipei, Taiwan). The mice were maintained in the pathogen-free facility of Change-Gung Memorial Hospital and used at 8–12 weeks of age. Experimental use of these mice was approved by Animal Care Committee of Chang-Gung Memorial Hospital (No. 2010033102). The institutional and/or licensing committee approved the experiments, including any relevant details and all experiments were performed in accordance with relevant guidelines and regulations.

### Cell lines

Murine and human cancer cell lines were used. Hepa1-6 (murine hepatoma, H-2^b^), EL4 (murine lymphoma, H-2^b^), Hep G2 (human hepatoma), 3B (human hepatoma), SK (human hepatoma), and Huh7 (human hepatoma) cell lines were obtained from the Cell Collection and Research Center (CCRC, ShinChu, Taiwan). The cells were maintained in Dulbecco’s minimal essential Medium (DMEM, Life Technologies, Gaithersburg, MD) and supplemented with 10% v/v FBS.

### Analysis of surface molecule expression

After the surface molecules were stained directly by PE-conjugated anti-Gr-1 monoclonal antibodies and FITC-conjugated anti-CD11b monoclonal antibody (1/100 × for rat monoclonal antibodies, and 1/50 × for mouse monoclonal antibodies; PharMingen, San Diego, CA, USA), the cell surface expression of markers on splenocytes was analyzed by flow cytometry employing a Coulter Epics Altra flow cytometer (Beckman Coulter Co., Miami, FL, USA). Isotype immunoglobulin was used as a control.

### Quantitation of cytokine production

The supernatant of Hepa1-6 cell culture and serum from mice with or without tumors was collected. SCF was measured by ELISA. The procedure was conducted using the instructions of the producer (PharMingen or R&D Systems Inc., Minneapolis, MN, USA).

### Conditioned medium

Conditioned medium was prepared by culturing Hepa1-6 cells in RPMI containing 5% FBS for 48 h in T-25 culture flasks. Cells were filtered from the medium using a 0.2 mm filter (Millipore, Bedford, MA, USA).

### Cytokine array

Supernatant of Hepa1-6 cell culture and serum from tumor-bearing mice were collected, incubated with cytokine antibody array membrane, and developed according to the manufacturer’s instructions. The cytokine antibody array membrane included mouse cytokine G3 and G4 (RayBiotech, Inc. Norcross, GA, USA).

### Propagation of bone marrow-derived DCs

To propagate bone marrow-derived DCs, bone marrow cells were harvested from femurs and tibias of normal B10 mice. The cells were cultured in 24-well plates (2 × 10^6^ cells/well) in 1 ml of RPMI-1640 medium (Life Technologies), supplemented with 4 ng/ml recombinant mouse GM-CSF (Schering-Plough Kenilworth, NJ) and 1,000 U/ml mouse interleukin-4 (R&D Systems Inc.). DCs were harvested after 5 days of culture. Prior to harvest, the DCs were pulsed with a Hepa1-6 lysate (1 × 10^5^ cell lysate/well) overnight.

### T-cell proliferation

T-cells were stained by 5-(and-6)-carboxyfluorescein diacetate succinimidyl ester according to the manufacturer’s instructions (Sigma-Aldrich, St. Louis, MO, USA) and stimulated by DCs. The proliferation of T-cells was determined by flow cytometry.

### Isolation of MDSCs

MDSCs were isolated using an MDSC isolation kit. The procedure was performed according to the manufacturer’s instructions (Miltenyi Biotec, Auburn, CA, USA).

### Protein preparation

The tumor cells were dissolved in lysis buffer containing 8 M urea, 4% CHAPS, and 65 mM dithiothreitol, and sonicated on ice for ten rounds of 10 s each. The final lysate was centrifuged for 1 h at 12,000 rpm to remove DNA, RNA and unsolved cell debris.

### Western blot

Proteins from Hepa1-6, EL4, Hep G2, 3B, SK, and Huh7 cells were separated on 12% polyacrylamide gels and transferred to polyvinylidene difluoride membranes (Amersham Biosciences, Uppsala, Sweden). These blots were incubated for 1 h at room temperature in Tris-buffered saline containing Tween (TBST; 20 mM Tris–Cl, 140 mM NaCl, pH 7.5, 0.05% Tween 20) also containing 5% skim milk. Primary antibody used was anti-SCF (diluted 1:1,000, Abcam, Cambridge, MA, USA). Blots were incubated with primary antibodies overnight at 4 °C. After washing three times in TBST, blots were incubated with horseradish peroxidase-conjugated secondary antibody (diluted 1:2000, Santa Cruz Biotechnology, Dallas, TX, USA) for 1 h at room temperature. Immunoreactive complexes were visualized using enhanced chemiluminescence reagents (Millipore, Billerica, MA, USA).

### Tumor inoculation

Hepa1-6 cells (5 × 10^5^) were subcutaneously inoculated in a flank of B10 mice to induce hepatoma. EL cells (1 × 10^5^) were subcutaneously inoculated in the flank of B6 mice to induce lymphoma. The tumors were allowed to grow until they were 10–15 mm in diameter. The tumor volume was calculated as 0.52 × width^2^ × length^39^. The mice were sacrificed when the diameter of tumor reached 20 mm.

### Arginase activity

Arginase activity was measured in cell lysates using an arginase assay kit (Abnova Corp., Taipei, Taiwan). Cells (2 × 10^6^) were lysed for 10 min in 100 μl of 0.1% Triton X-100. The cell lysate was centrifuged at 14,000*g* at 4 °C for 10 min. The supernatant was collected for the arginase assay performed according to the manufacturer’s instructions.

### Splenectomy

Under adequate anesthesia with isoflurane inhalation, an oblique incision was made over left flank of the mice to access the abdominal cavity. The spleen was located just beneath the wound and was pulled out. The splenic artery and vein were ligated and the spleen was removed. The wound was closed with 4-0 Dexon sutures.

### Excision of tumor

Under adequate anesthesia, an incision over the tumor was made. The well-defined tumor was freed from the surrounding tissue and removed. The wound was closed.

### Blocking of SCF

SCF shRNA was incorporated into the Block-IT™ pENTR/H1/TO vector. The sense sequence shRNA was CACCGCAGTCAAGTCTTACAAGGGCGAACCCTTGTAAGACTTGACTG and the antisense sequence was AAAACAGTCAAGTCTTACAAGGGTTCGCCCTTGTAAGACTTGACTGC. Hepa1-6 was transfected with shRNA of SCF using Lipofectamine (Invitrogen, Thermo Fisher Scientific, Waltham, MA, USA). Transfection was conducted as per the manufacturer’s instructions.

### Biostatistics

The comparisons of categorical variables were determined by Chi-square or Fisher’s Exact Tests if test number was < 5. The significance of the differences between different groups was determined by paired or unpaired Student’s t test. Survival rates were calculated using the Kaplan–Meier method and compared between groups using the log-rank test (SigmaStat 3.0 software). *P*-values < 0.05 were considered significantly different.
